# Twenty-five years of human metapneumovirus research

**DOI:** 10.1128/cmr.00330-25

**Published:** 2026-07-10

**Authors:** Elhadji Birane Mboup, Julia Dubois, Marie-Ève Hamelin, Manuel Rosa-Calatrava, Guy Boivin

**Affiliations:** 1 Infectious and Immune Diseases Research Program, CHU de Québec-Université Laval Research Center, Québec, Canada; 2 International Research Laboratory RESPIVIR France-Canada, Centre de Recherche en Infectiologie, Faculté de Médecine RTH Laennec, Université de Lyon, Université Claude Bernard Lyon 1, Lyon, France; 3 CHU de Québec-Université Laval Research Center, Québec, Quebec, Canada; 4 CIRI, Centre International de Recherche en Infectiologie, Team VirPath, Univ Lyon, Inserm, U1111, Université Claude Bernard Lyon 1, CNRS, UMR5308, ENS de Lyon, Lyon, France; 5 Department of Pediatrics, Faculty of Medicine, Université Laval 12369, Québec, Quebec, Canada; Institute for Hepatology, National Clinical Research Center for Infectious Disease, Shenzhen Third People's Hospital, Shenzhen, China

**Keywords:** human metapneumovirus, epidemiology, clinical features, host response, treatment, vaccines, respiratory syncytial virus

## Abstract

The year 2026 marks the 25th anniversary of the discovery of human metapneumovirus (HMPV), which belongs to the metapneumovirus genus within the *Pneumoviridae* family. The respiratory syncytial virus (RSV), discovered in 1956, is a close relative of HMPV belonging to the same family but to a different genus. In this article, we review key past findings related to HMPV infection, highlighting the differences with RSV. In addition, we specifically discuss current research areas pertaining to changing epidemiology, clinical burden, host response, as well as therapeutic and prophylactic modalities now in clinical trials. Additional research on HMPV is needed to contain this important respiratory virus.

## INTRODUCTION

**Fig 1:**
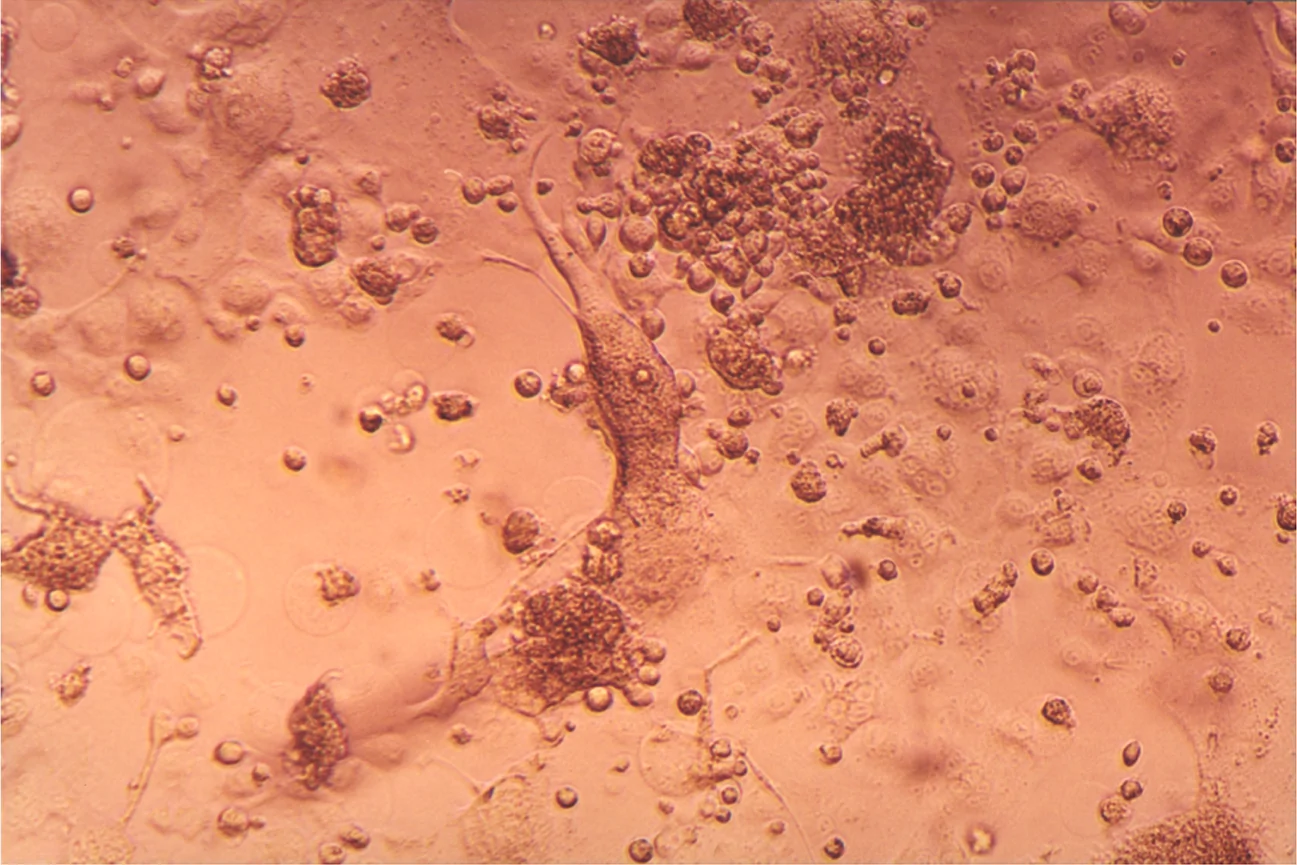
Cytopathic effects (syncytia) induced by HMPV on LLC-MK2 cells.

Human metapneumovirus (HMPV) was discovered in 2001 by a Dutch group led by Dr. Albert Osterhaus (1). This enveloped virus was initially isolated from nasopharyngeal aspirates of infants with bronchiolitis. Our group was the second to detect it and the first to report its presence in respiratory samples from all age groups (2). In fact, HMPV was not an emerging virus at that time but a long-lasting unidentified pathogen. Some of the reasons for its late identification include clinical features similar to those of the respiratory syncytial virus (RSV) and, more importantly, the long period of time (mean of 17 days) before appearance of cytopathic effects (mainly syncytia) in limited cell lines such as LLC-MK2 cells (Fig. 1) (2). Furthermore, serological evidence of infection was found using human sera collected in the 1950s (1) with retrospective positive RT-PCR assays of unidentified cell cultures from the 1980s (2). The virus was initially classified in the *Paramyxoviridae* family and then re-classified in 2016 in the newly created *Pneumoviridae* family along with RSV (3). In the same family, there is also the avian metapneumovirus (AMPV) that is responsible for laryngotracheitis in poultry. Using molecular clock studies, HMPV likely diverged from AMPV, most especially subtype C, around 200 years ago (4), giving rise to two major genetic lineages A and B (5). Structurally, HMPV shares the typical architecture of pneumoviruses, forming spherical pleomorphic-shaped virions with a diameter of roughly 150–200 nm and densely decorated with surface glycoproteins, which are essential for cell entry and immune evasion (6).

**Fig 2:**
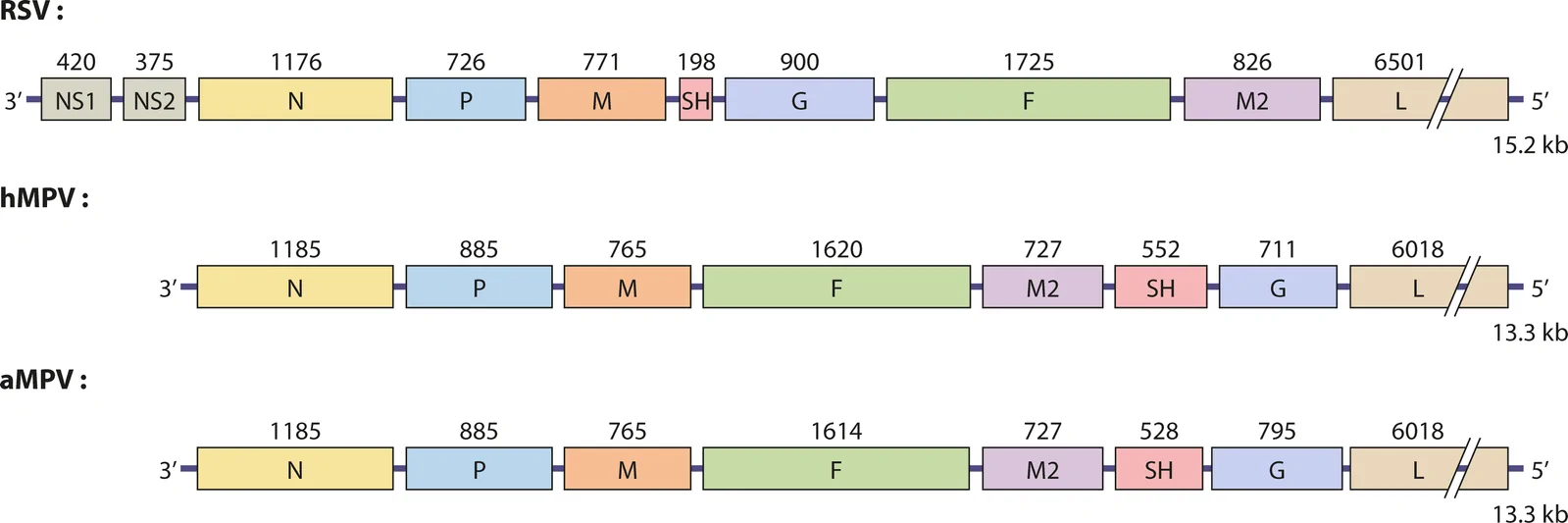
Comparison of viral genomes of pneumoviruses (RSV, HMPV, and avian metapneumovirus [AMPV]); numbers above the bars indicate nucleotide base pairs.

The genome of HMPV consists of a single-stranded, negative polarity, RNA molecule of 13.3 kb coding for nine proteins (Nucleoprotein, N; Phosphoprotein, P; Matrix protein, M; Fusion glycoprotein, F; M2-1 and M2-2 proteins; Small Hydrophobic protein, SH; Attachment protein, G; Large protein/polymerase, L) (6). On the other hand, the RSV genome is longer (15.2 kb) due to two additional genes (NS1 and NS2) coding for key modulators of host immunity (7). Also, the order of the other shared genes differs between the two pneumoviruses (5) (Fig. 2).

HMPV and RSV follow a broadly similar replication cycle. After attachment to respiratory epithelial cells via their G and/or F glycoproteins, the F protein mediates fusion of the viral envelope with the host cell membrane, releasing the ribonucleoprotein complex into the cytoplasm. The viral RNA-dependent RNA polymerase (L, with co-factor P) transcribes the genomic RNA into capped and polyadenylated mRNAs, which are translated into viral proteins. As N protein accumulates, the polymerase switches from transcription to replication, synthesizing full-length antigenomes and then progeny genomes encapsidated by the N protein. Newly formed nucleocapsids associate with M at the plasma membrane, where F, G, and SH glycoproteins have been inserted into the host-derived lipid bilayer, leading to assembly and budding of mature virions that are released to infect neighboring cells (8).

Both HMPV and RSV have evolved sophisticated strategies to blunt type I/III interferon responses and distort T-cell immunity, while using partly distinct molecular tools. RSV relies mainly on NS1/NS2, with G and SH further modulating chemokines, NF-κB, and inflammasomes. In contrast, HMPV, which lacks NS1/NS2, uses G, SH, and M2-2 to inhibit TLR4- and MAVS-dependent signaling, dampen cytokine induction, and impair dendritic cell and T-cell activation, promoting a Th2-skewed, poorly protective response (9–11). These convergent yet mechanistically distinct evasion strategies help both viruses to persist in the population, despite widespread exposure in early childhood, complicating vaccine and antiviral design.

Current research questions and differences between HMPV and RSV are discussed under the points listed below.

## THE CHANGING EPIDEMIOLOGY OF HMPV

The large outbreak of HMPV in China in 2024–25 (due to a re-occurrence of the B2 genotype and not a novel one) reminds us of the preponderant role of this pathogen spreading by infectious airborne droplets, like other respiratory viruses (12). Based on an international collaborative study, our group first reported the classification of HMPV strains into four genotypes (A1, A2, B1, and B2) in 2003–04 by analyzing G or F gene sequences (5, 13). Subsequently, the A2 genotype was divided into two other ones, i.e., A2a and A2b (14). Ongoing evolution mainly within the A2 lineage revealed another sublineage: A2c. Recent studies have reported the predominance of the A2b genotype with the disappearance of subtype A1 since 2006 (15, 16). In addition, new sublineages with duplications in the G gene have been reported (15). Importantly, despite such genetic diversity, HMPV is reported to have only one serotype (17). An important research question is whether some genotypes are associated with a more severe outcome. In this respect, we reported that HMPV genotype B was independently associated with a more severe outcome in young children during a 4-year-long study (18). Conversely, others have reported an association between a worse clinical outcome and HMPV genotype A infections (19) or no significant association (20). Thus, additional studies on this topic should be performed including viral genotyping, specific age groups, co-morbidity factors, co-infections, and patients’ putative genetic and immunity defects (21).

Different genotypes can circulate simultaneously in the same geographical area although one strain typically dominates. Conversely, different strains may circulate each year in different countries (13, 22). All children are infected by the age of 5–10 years, although reinfections can occur throughout life, due to short-lived immunity and the diversity of viral strains (13, 23). The median age of HMPV-positive hospitalized patients is 6–12 months, which is higher than that of RSV (2–3 months) (24, 25). Such a delay in HMPV susceptibility may have an impact on the development of vaccines and monoclonal antibodies against the two pneumoviruses. Globally, in 2018, HMPV was estimated to cause 502,000 hospitalizations and 11,300 deaths in young children (below 5 years) with a burden between that of RSV and influenza (26). In older adults (above 65 years), HMPV-associated hospitalization rates (231/100,000) are almost the same as for RSV (350/100,000) (27).

In the northern hemisphere, HMPV circulates predominantly in the late winter and spring seasons; the HMPV peak occurring systematically after the RSV one in temperate countries from both hemispheres although there is an overlap in the circulation of the two viruses (28). In a 10-year US surveillance study, the interval from peak RSV (late December) to peak HMPV (late March) was 11.5 weeks (29). This interval increased to 19 weeks during and after the COVID-19 pandemic. Interestingly, at the global level, HMPV appears to circulate in a counterclockwise manner, whereas RSV seems to circulate in a clockwise manner during 2022–24 (30).

The difference in the two peaks of virus circulation could be explained, in part, by the concept of viral interference between respiratory viruses (31) and trained immunity (32). In a collaborative study with a Swiss group, we showed in a model of reconstituted human airway epithelium that HMPV replication was blocked by a prior RSV infection 2 days earlier, whereas RSV was not affected by earlier HMPV infection (33). Further studies are required to see whether such interaction could persist beyond the period of active viral replication.

Finally, a shift in viruses associated with bronchiolitis/pneumonia could potentially occur with the availability of potent monoclonal antibodies for RSV. In a study looking at viral etiologies in children hospitalized with lower respiratory tract infections (LRTI) pre- and post-Nirsevimab prophylaxis, RSV infections were significantly reduced, while HMPV infections were significantly on the rise (34). Additional information is required to confirm the concept of viral/bacterial replacement following successful strategies to prevent or delay RSV infections. Notably, the role of Next Generation Sequencing and Whole Genome Sequencing will become crucial for HMPV genome analysis following the introduction of monoclonal antibodies (mAbs) and vaccines as shown for RSV (35, 36).

## THE (UN) DISTINGUISHING CLINICAL FEATURES OF HMPV AND RSV

From a clinical point of view, HMPV shares many overlapping features with RSV across age groups, to the point that they are often difficult to distinguish without virological testing. They cause a broad spectrum of acute respiratory illnesses (ARI), ranging from mild upper respiratory tract infections (URTI) to more severe LRTI in children or the elderly. Clinical presentation can include fever, cough, dyspnea, tachypnea, or viral wheeze leading to the diagnosis of bronchitis or bronchiolitis, pneumonia, decompensated chronic heart disease, or, in more severe cases, acute respiratory failure (37). Examination and chest x-rays of hospitalized patients can help to differentiate between HMPV- and RSV-associated ARI despite similar severity and supportive ventilatory requirements (23, 38). Typical bronchiolitis characterized by chest retractions, widespread wheeze, prolonged expiratory phase, hyperinflation, and diffuse perihilar infiltration is more classically associated with RSV infection (23). In contrast, children hospitalized with HMPV are less likely to present with wheezing than those infected by RSV but more frequently with fever, cough, crackles, and focal or diffuse alveolar infiltrates on chest x-rays, which are characteristic of viral pneumonia or pneumonitis.

HMPV- and RSV-associated ARI constitute an accountable part of outpatient visits during the winter and early spring, and severe LRTIs in at-risk populations represent an important burden in hospitals, especially in pediatric intensive care units, concomitantly with the influenza season. Recurrently noticed, viral-viral and viral-bacterial co-infections can also contribute to severity, non-invasive ventilation failure, and fatality, regardless of whether HMPV or RSV is responsible for the initial infection, in vulnerable populations like children, elderly, and immunocompromised patients (39). HMPV still remains under-detected in both outpatients and inpatients with ARI, partly because RSV has been included for a longer time in multiplex diagnostic panels and rapid antigenic tests. However, surveillance studies consistently show that the median age of children hospitalized with RSV is lower than that of those with HMPV infections. In large pediatric cohorts, children hospitalized with RSV tend to be younger, with a peak of severe disease in infants below 6–12 months, whereas HMPV cases are on average older, more frequently in children from 6 to 24 months, up to 5 years old (38, 40–43). Overall outcome differences between HMPV and RSV remain modest, and the risk of severe illness caused by these two viruses is increased by comorbidities, such as asthma and chronic obstructive pulmonary disease (COPD), co-infections, or other cardiovascular, autoimmune, or metabolic pathologies (39, 43). History of prematurity also accounts for a high-risk factor of severity of HMPV and RSV infections for more than 20% of hospitalized children. To note, HMPV-infected children admitted to intensive care are more likely to have underlying comorbidities, congenital heart disease, chronic lung disease, or neuromuscular conditions, with a slightly more severe disease course (44), while RSV has a higher impact in otherwise healthy young infants (43).

In adults and those with comorbidities, HMPV and RSV infections frequently present as non-specific “viral pneumonia” or exacerbations of underlying COPD, asthma, or heart failure, making etiological discrimination based on clinical grounds alone particularly unreliable (45, 46). Several cohort studies and reviews, therefore, emphasize that syndromic case definitions cannot separate HMPV from RSV infections in routine clinical practice (37, 38). Among HMPV- and RSV-infected adult patients, age and at least one comorbidity are the major risk factors for hospitalization although HMPV seems to occur less likely in smokers or ex-tobacco users than RSV (45). Lower respiratory illnesses caused by HMPV infections are also more frequent in immunocompromised (children and adults) hosts and in the elderly population (over 65-years-old). These populations are especially vulnerable and develop symptomatic to severe pathologies such as acute respiratory distress syndrome, respiratory failure, and aggravating preexisting conditions (chronic respiratory disease, cardiac disease) (46). In high-risk adults, half of medically attended HMPV cases require hospitalization, with about 6% being admitted in intensive care units resulting in a fatality rate of 4%, a proportion similar to that of RSV (47). Cases of HMPV-infections in individuals with hematological malignancies or solid tumors, undergoing chemotherapy, hematopoietic stem cell transplant, or lung transplant, are reported with an increased fatality rate, given the lack of therapeutic arsenal and limitation of interventional procedures.

The overall clinical overlap between RSV and HMPV, despite some subtle distinguishing features, highlights that bedside assessment alone cannot reliably differentiate between these infections, notably in children and vulnerable adults. In practice, etiological diagnosis relying on laboratory testing should include HMPV as essential target for accurate surveillance, infection control, and faster patient care (48, 49). Accordingly, several multiplex PCR panels now include HMPV among different respiratory pathogens of interest (influenza A and B viruses, rhinoviruses, parainfluenza viruses, adenoviruses, coronaviruses, and RSV) and offer rapid and sensitive virus screening in both hospital and community settings (50) which significantly improve clinical diagnosis and control-practice of pneumovirus infections.

## IMMUNE CROSS-REACTIVITY BETWEEN HMPV AND RSV AND DEVELOPMENT OF MONOCLONAL ANTIBODIES

Immune cross-reactivity between subgroups of HMPV and, less frequently, between HMPV and RSV, including cross-binding antibodies, cross-neutralizing antibodies, or cross-reactive T cells, has been documented with direct implications for reinfection patterns, age-specific disease burden, and rational vaccine design (51, 52). Despite only 30%–35% of amino acid identity with RSV F, the HMPV F protein was initially described as having six major antigenic sites (Ø–V), named according to the RSV nomenclature, but mapping with highly HMPV-neutralizing monoclonal antibodies has then revealed additional overlapping and distinct epitopes from the RSV F protein. Unlike RSV, whose most potent neutralizing antibodies are strongly pre-fusion F-biased, HMPV-neutralizing antibodies generally recognize both pre- and post-fusion conformations, and pre-F-specific antibodies show only weak or undetectable HMPV neutralization (53). This difference can be explained by the blocking of the site Ø of the HMPV F protein, as the apex of the pre-fusion conformation head, by glycosylation sites (54). Consequently, the discovery of monoclonal antibodies that are both potently neutralizing and broadly cross-reactive against RSV and HMPV has proven to be particularly challenging (51).

The HMPV-specific B cell repertoires of healthy seropositive or convalescent patients have been shown to be highly divergent among donors, partly explained by the high frequency of repeated infections throughout their lifetime, and only a minority (less than 3.5%) displayed additional reactivity against RSV F glycoprotein (53, 55). Among hundreds of HMPV-specific mAbs identified, some of them targeting in or near antigenic sites III or IV exhibited broad activity against both HMPV and RSV, highlighting the potential of non–pre-F-biased antibodies such as MP8 (56), 54G10 (57), RSV-199 (58), and MxR (59) (Table 1). Administration of these mAbs in permissive animal models has shown meaningful therapeutic efficacy when given after viral challenge, with notably systemic or intranasal delivery of 54G10 reducing infection-associated mortality, lowering viral loads—often more effectively in the lower than in the upper respiratory tract—and limiting pulmonary inflammation. Interestingly, another candidate (mAb 100) that we evaluated substantially decreased HMPV and RSV titers in mice, despite relatively modest *in vitro* neutralization, suggesting that additional effector mechanisms beyond standard Fab-mediated neutralization readouts should contribute to *in vivo* protection (60). Recently, another cross-reactive mAb (CNR2047) has been identified, binding to a conserved epitope in site III across HMPV and RSV, with a similar mode than MP8, RSV-199, or MxR (61). Looking further into the structure of site III-directed antibodies, specificities in Heavy Chain Complementary-Determining Region 3 (HCDR3) have been identified that confer a flexible fitting into a cavity formed between two monomers of HMPV F or RSV F protein. The authors also showed the *in vivo* efficacy of a cocktail of CNR2047 with RSV-specific mAb (binding to the Ø site), providing complete protection in the lungs of animals challenged with RSV-A, RSV-B, or HMPV-A. Investigating the binding of this combination of mAbs onto F proteins, they suggested that III- and Ø-directed mAbs combination could provide broad protection, acting as a shield blocking the viral attachment and facilitating the disassembly of pre-F trimers, highlighting the advantage to use a mix of non-competing and cooperative mAbs to confer a “pan-pneumovirus” protection.

**TABLE 1:** Monoclonal HMPV-specific or HMPV-RSV cross-reactive antibodies of interest^*a*^

Name	Screen F antigen	Target HMPV epitope	Cross-neutralizing	*In vitro* neutralization titers (IC50, ng/mL)	*In vitro* binding efficacy (EC50, ng/mL)	Efficacy in animal models	Ref.
DS7	HMPV-A2	I	NA	2,400 (HMPV-A1), 1,100 (HMPV-A2), 9,800 (HMPV-B1)	13.5 (HMPV)	>1,500-fold HMPV titer reduction in lungs (therapeutics, cotton rat)	(55, 62, 63)
338	HMPV-A1	II	NA	380 (HMPV-A1), 800 (HMPV-A2), 30 (HMPV-B1), 170 (HMPV-B2)	NA	Reduction of HMPV lung replication and histopathology (prophylaxis, mouse, and hamster); efficacy when used in therapeutics (mouse)	(64, 65)
MP8	HMPV-A1; RSV-A	III (pre-F)	Yes	28 (HMPV-A1), 11 (HMPV-A2), 22 (HMPV-B1), 110 (HMPV-B2), 30 (RSV-A), 7.5 (RSV-B)	30 (HMPV preF), 2 (RSV)	Dose-dependent protection against lethality and reduction of lung RSV-A2 and HMPV-A titers (prophylaxis, mouse)	(56, 58)
101F	RSV-A	IV	Yes	5 (HMPV-A2), 770 (HMPV-B2), 675 (RSV-A), 970 (RSV-B)	NA	NA	(66–68)
54G10	HMPV-B2	IV	Yes	90 (HMPV-A1), 400 (HMPV-A2), 210 (HMPV-B1), 60 (HMPV-B2), 14,200 (RSV-A)	NA	Dose-dependent reduction of lung HMPV-A1, -B1, and -B2 and RSV-A2 titers and reduction of lung inflammation (prophylaxis, mouse); reduction of HMPV-A2 and RSV-A2 titers after therapeutic intranasal or intraperitoneal administration (mouse)	(57)
ADI-61026	HMPV-A1 (preF) ; HMPV-B2	Ø	No	39 (HMPV-A1), 14 (HMPV-A2), 15 (HMPV-B1), 1 (HMPV-B2)	NA	10- to 20-fold decrease in viral mRNA level in lungs; >100-fold decrease in HMPV viral titer (prophylaxis, cotton rat)	(53)
ADI-61104	HMPV-A1 ; HMPV-B2	I and III	No	41 (HMPV-A1), 36 (HMPV-A2), 28 (HMPV-B1), 2 (HMPV-B2)	NA	10- to 20-fold decrease in viral mRNA level in lungs; >100-fold decrease in HMPV viral titer (prophylaxis, cotton rat)	(53)
ADI-61556	HMPV-A1; HMPV-B2	II and III	No	12 (HMPV-A1), 25 (HMPV-A2), 37 (HMPV-B1), 22 (HMPV-B2)	NA	10- to 20-fold decrease in viral mRNA level in lungs; >100-fold decrease in HMPV viral titer (prophylaxis, cotton rat)	(53)
mab 82	HMPV-A1	NA	No	13.9 (HMPV-A), 1.7 (HMPV-B)	4.4-6.5 (HMPV)	>1,000-fold decrease in HMPV lung titer; negligible for RSV (prophylaxis, mouse)	(60)
mab 83	HMPV-A1	NA	No	17.2 (HMPV-A), 9.5 (HMPV-B)	4.4-6.5 (HMPV)	>1,000-fold decrease in HMPV lung titer; negligible for RSV (prophylaxis, mouse)	(60)
RSV-199	RSV-A2	III	Yes	43 (HMPV-A2), 8–63 (HMPV-B2), 14–54 (RSV-A), 4–12 (RSV-B)	2 (HMPV and RSV preF)	Dose-dependent reduction of lung RSV-A, RSV-B, and HMPV-A titers (prophylaxis, cotton rat)	(58)
mAb 100	HMPV-A1	NA	Yes	1,000 (HMPV-A), 10,000 (HMPV-B)	4.4-6.5 (HMPV)	>1,000-fold decrease in HMPV lung titer; >100-fold decrease in RSV lung titer (prophylaxis, mouse)	(60)
MxR	HMPV and RSV preF	III	Yes	148 (HMPV), 256 (RSV-A)	NA	>1,000-fold RSV titer reduction in lungs; 47-fold RSV reduction in nasal turbinates; >206-fold HMPV reduction in lungs (prophylaxis, hamster)	(59)
CNR2047	RSV preF	III	Yes	28.7 (HMPV-A1), 8.9–9.7 (RSV-A), 17.6–18.3 (RSV-B)	11.7 (RSV-pre-F)	>100-fold HMPV titer reduction in lungs at 0.5 mg/kg (prophylaxis, mice); 100-fold RSV-A and RSV-B reduction in lungs at 1 mg/kg (prophylaxis, cotton rats); lesser histopathological damages at higher doses	(61)

^*a*^ NA, not available.

To date, no HMPV-targeted mAb has advanced to phase 3 clinical trials, and no dual RSV/HMPV cross-protective mAb has progressed into late-stage clinical evaluation. The identification of conserved cross-neutralizing epitopes on the F protein has catalyzed the concept of “pan-pneumovirus” vaccine or immuno-prophylaxis. However, gaps remain in the understanding of the mechanisms of cross-reactivity—like Fc-mediated mechanisms such as antibody-dependent cellular cytotoxicity or antibody-dependent virus opsonization—and the magnitude and durability of cross-reactive responses depending on age groups, immune status, and exposure history. Also, infections with RSV and HMPV have been associated with aberrant or dysregulated T-cell responses, including Th2/Treg skewing and suboptimal CD8 memory (69, 70), a pattern thought to impair viral clearance and favor recurrent infections with either virus despite prior exposure. Thus, future efforts should be devoted to investigating the RSV-HMPV cross-reactivity more systematically at both the B- and T-cell levels in longitudinal human studies, alongside vaccine and monoclonal antibody trials. In these trials, it will also be important to monitor for the emergence of escape mutation, especially at the site Ø (71), that could impact the efficacy of prevention strategies including antibody prophylaxis and vaccination. Deep learning and structure-guided antigen design focusing on multi-conserved or cross-reactive epitopes (72) could also lead to new cross-protective treatment against RSV and HMPV in high-risk populations.

## WHO WOULD BENEFIT FROM A TREATMENT AGAINST HMPV AND WHAT ARE THE OPTIONS?

Treatment of severe HMPV infections essentially relies on supportive care such as oxygen supplementation, hydration, and antipyretic administration (73). To date, there are no antivirals or monoclonal antibodies in the clinic and the only therapeutic agent in clinical trial is ALVR106 (NCT04933968), a multi-virus-specific T lymphocyte therapy for patients suffering from respiratory infections following hematopoietic stem cell and solid organ transplant. Viral clearance by day 28 suggests a potential efficacy for T-cell therapy against severe HMPV infections (74). Hence, there is still a need to find molecules for at-risk populations such as immunocompromised individuals—who might not gain complete protection from eventual vaccination—premature infants, young children, and the elderly.

Several candidates with proven efficacy against other respiratory viruses, including RSV, have been repurposed as potential candidates for HMPV. Their main mechanisms of action are polymerase inhibition (ribavirin, favipiravir, and MRK-1), entry/release inhibition (heparin, argantroban, warfarin, RWJ-56110, suranim, γ-Fagarin, ginkgolic acid, DAS181, etc.), fusion inhibition (synthetic peptides mimicking fusion protein’s heptad repeat regions HRA and HRB), and cellular kinase inhibition (probenecid, IC87114, BX795, ruxolitinib, fumarprotocetraric acid, and geraniin) (75, 76). Ribavirin, a guanosine analog effective against RSV, is also administered by nebulization or by the systemic route in HMPV infection as a last-resort therapy to immunocompromised patients such as stem cell transplant recipients with LRTI, sometimes in combination with intravenous immunoglobulin G (IVIG) for enhanced efficacy (77–79). However, inconsistent efficacy and reported side effects, including teratogenicity, limit its use in humans (80, 81).

## DEVELOPMENT OF HMPV VACCINES: LESSONS LEARNED FROM RSV VACCINES

Since HMPV was described over 40 years after RSV, vaccine development has been mainly focused on the latter. The first RSV vaccine tested in humans, a formalin-inactivated formulation, failed to confer protection and also induced enhanced respiratory disease (ERD) in vaccinees and reports of lethal cases (82). Research on the stabilization of the antigenic F protein in a prefusion conformation enabled the rational design of PreF-based RSV vaccines, including two licensed subunit vaccines ABRYSVO (Pfizer) and AREXVY (GSK) and one mRNA vaccine mRESVIA (Moderna). Such vaccines are primarily indicated for active immunization in older adults and pregnant women (ABRYSVO only) and have proven their efficacy against RSV-related LRTI and hospitalizations (83–85).

As mentioned earlier, antibodies against epitopes located at overlapping antigenic sites have also been identified, and most anti-HMPV F antibodies target epitopes across both protein conformations, highlighting the immunogenicity diversity of the F antigens between the two viruses (51). A prefusion-stabilized HMPV F protein (DS-CavEs2) has been shown to elicit higher neutralizing antibody titers for genotype A1 and B1 than the post-fusion conformation (86). However, it has also been demonstrated that there is no predominance of the prefusion conformation over the post-fusion regarding HMPV immunization (87), as well as against wild-type or codon-optimized vectored versions of the F antigen (88).

Although licensed vaccines remain unavailable for HMPV, lessons learned from RSV vaccine development provided a roadmap for HMPV vaccine research. Inactivated HMPV vaccines have been tested in animal models (mice and cotton rats), where ERD was observed following viral challenge. A Th2-biased immune response was reported, highlighting the risk associated with inactivated HMPV vaccines (89, 90). Therefore, to mitigate fatal outcomes in humans, alternate safer platforms are being privileged for vaccine development against this pathogen (Table 2).

**TABLE 2:** HMPV vaccine candidates in clinical trials

Candidate	Sponsor	Platform	Target	Status
rHMPV-Pa	NIAID	Live-attenuated	HMPV	Discontinued
B/HPIV3/HMPV-PreF-A			HMPV + HPIV3	Phase 1
B/HPIV3/HMPV-F-B365				
mRNA-1365	Moderna	mRNA	HMPV + RSV	Paused
mRNA-1653			HMPV + HPIV3	Phase 1
SP0256	Sanofi		HMPV + RSV	Phase 2
SP0291			HMPV + RSV + HPIV3	Phase 1
IVX-A12	Icosavax	VLP	HMPV + RSV	Phase 2a
VXB-241	Vicebio	Subunit (molecular clamp)	HMPV + RSV	Phase 1
VXB-251			HMPV + RSV + HPIV3	
SCB-1022	Clover Biopharmaceuticals	Subunit (Trimer-Tag)	HMPV + RSV	Phase 2
SCB-1033			HMPV + RSV + HPIV3	

The first clinical trial on an HMPV vaccine assessed a recombinant live-attenuated strain rHMPV-Pa, where the P gene was replaced by the one from AMPV. The study was discontinued due to an over-attenuated profile in seronegative children (91). Lately, because of similar risk factors, clinical manifestations, and populations susceptible to HMPV, RSV, and HPIV3 infections, combination vaccines targeting these viruses have been prioritized, and several candidates are in the clinical pipeline.

The mRNA technology offers a flexible platform for designing combined vaccines. mRNA-1653 targeting HMPV and HPIV3 and mRNA-1365 targeting RSV and HMPV are two bivalent candidates developed by Moderna. mRNA-1653 demonstrated an acceptable safety profile in adult and seropositive children and also boosted neutralizing antibody (nAb) titers for both viruses (geometric mean fold-rise ratio over baseline: HMPV/A = 2.9–6.1, HMPV/B = 6.2–13.2, PIV3 = 2.8–3.0), with pre-F biased binding antibody response (92, 93). For the evaluation of mRNA-1365, investigators reported an increase of RSV nAbs in children aged 8–23 months, with *de novo* nAb responses in RSV-naïve infants 5–7 months old. Moreover, vaccination induced a preF-biased binding antibody response in both age groups and RSV-specific Th1 responses in older children. Unfortunately, the study was suspended following the occurrence of severe RSV-LRTI in some participants, suggesting vaccine-associated ERD (94).

Sanofi has launched trials assessing bivalent SP0256 (RSV/HMPV) and trivalent SP0291 (RSV/HMPV/HPIV3), both mRNA-based candidates (NCT06583031, NCT06237296, NCT06604767). The company has recently expanded its pipeline beyond mRNA by acquiring Vicebio, whose pipeline includes two candidates currently undergoing phase 1 trials: VXB-241, a bivalent vaccine targeting RSV and HMPV (NCT06556147), and VXB-251, a trivalent candidate targeting RSV, HMPV, and HPIV3 (NCT07295028). These subunit candidates are based on stabilized antigens, developed with Vicebio’s molecular clamp technology (95).

Novel-stabilized candidates have been introduced by Clover Biopharmaceuticals. SCB-1022 (RSV/HMPV) and SCB-1033 (RSV/HMPV/HPIV3) are combination vaccines currently in phase 2 (96). They are prefusion trimer subunit antigens (Pre-Trimer), stabilized with Clover’s Trimer-Tag vaccine technology platform (97).

Another subunit candidate was developed by Icosavax. IVX-A12 is AstraZeneca’s bivalent RSV/HMPV candidate, featuring virus-like particles (VLP) of pre-F stabilized antigens from both viruses. In a phase 1 trial in adults of 60–75 years of age, this candidate boosted RSV- and HMPV-specific neutralizing antibodies at all dose levels, regardless of the addition of an adjuvant (98).

Finally, two candidates based on a live-attenuated platform are sponsored by NIAID. B/HPIV3/HMPV-PreF-A and B/HPIV3/HMPV-F-B365 are Bovine/Human Parainfluenza Virus Type 3 (B/HPIV3) vectored vaccines expressing the fusion proteins of HMPV. These experimental candidates are intended to protect against HMPV and HPIV3, and a phase 1 trial assessing their safety in HPIV3-seropositive children is ongoing (NCT06546423).

## CONCLUSIONS

HMPV and RSV belong to the same *Pneumoviridae* family, yet many distinguishing features have been reported regarding their genomic organization, antigenic architecture, epidemiology, and interaction with host immunity (summarized in Table 3) that must now be fully integrated into prevention and treatment strategies. For instance, different peaks of circulating activity, age at first hospitalization, and F antigenic sites exist for HMPV and RSV, which should impact the timing of passive immunization as well as vaccine composition. Both fundamental and clinical/epidemiological research over the past 25 years have contributed to a better knowledge about HMPV, emerging from a “newcomer” in 2001, to a globally recognized cause of upper/lower respiratory tract infections in all age groups, as we initially reported in 2002 (2).

**TABLE 3:** Head-to-head comparison of HMPV and RSV key aspects

	HMPV	RSV
Genomic organization	Linear negative RNA genome 8 genes encoding 9 proteins 3′-N-P-M-F-M2-1/2-SH-G-L-5′	Linear negative RNA genome 10 genes encoding 11 proteins 3′-NS1-NS2-N-P-M-SH-G-F-M2-1/2-L-5′
Protein composition/functions	*Structural proteins:* Nucleoprotein, N; Matrix protein, M *RNA Polymerase complex:* Large protein/Polymerase, L; Phosphoprotein, P *Regulators:* M2-1 Antitermination protein; M2-2 Transcription/replication regulation protein *Surface glycoproteins:* Fusion glycoprotein, F; Small Hydrophobic protein, SH; Attachment protein, G	
		*Regulators of host immune response:* Nonstructural proteins, NS1 and NS2
Epidemiological features	– Four genotypes (A1, A2, B1, B2) – Predominance of A2 and B2 genotype circulation, peaks in March – 100% seropositivity rate by 5–10 years of age – Median age of hospitalization 6–12 months – Recurrent reinfections throughout life	– Co-circulation of genotypes A and B, peaks in late December; – 100% seropositivity rate by 5 years of age – Median age of hospitalization 2–3 months – Recurrent reinfections throughout life
Clinical manifestations	– *Clinical presentation:* Mild upper respiratory tract infections to severe lower respiratory tract infections in children, at-risk adults with co-morbidities and the elderly; symptoms include fever, cough, dyspnea, tachypnea, or viral wheeze – *Risk factors of hospitalization:* prematurity and age, cardiac disease, respiratory conditions (COPD, asthma), immunosuppression – *Diagnosis:* bronchitis or bronchiolitis, pneumonia, decompensated chronic heart disease, acute respiratory failure	
	More likely, viral pneumonia or pneumonitis characterized by fever, cough, crackles, and focal or diffuse alveolar infiltrates (x-ray)	More likely, typical bronchiolitis characterized by chest retractions, wheezing, prolonged expiratory phase and hyperinflation and diffuse perihilar infiltration (x-ray)
Vaccines and prophylactics	*Multivalent candidate in clinical trials:* – Phase 2: VLP vaccine HMPV + RSV (Icosavax); mRNA HMPV + RSV (Sanofi); HMPV + RSV ± HPIV3 (Clover Bioph.) – Phase 1: LAV HMPV + HPIV3 (NIAID); mRNA HMPV + RSV + HPIV3 (Sanofi), HMPV + HPIV3 (Moderna); subunits HMPV + RSV ± HPIV3 (Vicebio)	*Licensed vaccines:* For elderly: Adj. subunit (AREXVY, GSK), unadj. ---Subunit (ABRYSVO, Pfizer), Mrn-A (mRESVIA, Moderna) For pregnant women (passive immunization of newborns): unadj. subunit (ABRYSVO, Pfizer) *Licensed monoclonal antibodies (passive immunization of newborns):* Palivizumab (Synagis, AstraZeneca), Nirsevimab (Beyfortus, AstraZeneca), clesrovimab-cfor (Enflonsia, Merck)
Therapeutics	Supportive oxygen supplementation, hydration, and antipyretic treatment	
	Ribavirin + IVIG (last-resort therapy) *Clinical trial:* Phase 1–2: multi-virus-specific T lymphocyte therapy (hematopoietic stem cell transplant recipients)	Ribavirin (aerosol, approved for severe RSV infection but no longer recommended) *Clinical trials:* Phase 1–2: numerous antivirals targeting RSV proteins F (entry/fusion inhibitors), L or N (99) (replication inhibitors) Success in phase 3: Ziresovir (100)

As RSV vaccines and monoclonal antibodies are deployed and HMPV-specific candidates currently progress in their clinical evaluation, robust laboratory diagnosis—especially multiplex RT-PCR—will be essential for accurate surveillance, infection control, and attributing changes in disease burden to pathogen-specific or cross-pathogen effects. A major recent development is the establishment of a controlled human infection model (CHIM) for HMPV by hVIVO, using a well-characterized challenge HMPV strain to reproducibly induce mild-to-moderate upper respiratory infections in healthy adults under close safety monitoring (101). Such an HMPV CHIM model enables accelerated clinical proof-of-concept and derisking large field trials while offering a unique platform to dissect human correlates of protection, compare candidate regimens head-to-head, and generate translational data that can be integrated with RSV challenge studies to inform future pan-pneumovirus strategies. Key outstanding questions on HMPV and future research directions are summarized in Box 1.

Box 1. **Key outstanding questions on HMPV and future research directions**

Clarify the drivers of HMPV epidemiology.

Define the role of HMPV genotypes in disease severity.

Identify interference mechanisms between HMPV and other respiratory viruses.

Define HMPV-specific clinical features and high-risk groups.

Characterize cross-reactive immunity (with RSV) and protective epitopes.

Investigate immune evasion mechanisms.

Optimize therapeutic modalities for HMPV high-risk groups.

Understand pre- and post-fusion conformations and HMPV F antigenic sites.

Define the role of mucosal immunity for HMPV.

Advance and deploy effective HMPV and pan-pneumovirus vaccines.

The next decade will offer an opportunity to move beyond virus-specific strategies and toward rationally designed, cross-protective interventions to ultimately achieve durable, dual protection against severe pneumovirus diseases across the lifespan.

## References

[1] van den Hoogen BG, de Jong JC, Groen J, Kuiken T, de Groot R, Fouchier RA, Osterhaus AD. 2001. A newly discovered human pneumovirus isolated from young children with respiratory tract disease. Nat Med 7:719–724. doi:10.1038/89098 11385510 PMC7095854

[2] Boivin G, Abed Y, Pelletier G, Ruel L, Moisan D, Côté S, Peret TCT, Erdman DD, Anderson LJ. 2002. Virological features and clinical manifestations associated with human metapneumovirus: a new paramyxovirus responsible for acute respiratory-tract infections in all age groups. J Infect Dis 186:1330–1334. doi:10.1086/344319 12402203

[3] Afonso CL, Amarasinghe GK, Bányai K, Bào Y, Basler CF, Bavari S, Bejerman N, Blasdell KR, Briand F-X, Briese T, et al.. 2016. Taxonomy of the order *Mononegavirales*: update 2016. Arch Virol 161:2351–2360. doi:10.1007/s00705-016-2880-1 27216929 PMC4947412

[4] de Graaf M, Osterhaus A, Fouchier RAM, Holmes EC. 2008. Evolutionary dynamics of human and avian metapneumoviruses. J Gen Virol 89:2933–2942. doi:10.1099/vir.0.2008/006957-0 19008378

[5] Biacchesi S, Skiadopoulos MH, Boivin G, Hanson CT, Murphy BR, Collins PL, Buchholz UJ. 2003. Genetic diversity between human metapneumovirus subgroups. Virology 315:1–9. doi:10.1016/s0042-6822(03)00528-2 14592754

[6] Feuillet F, Lina B, Rosa-Calatrava M, Boivin G. 2012. Ten years of human metapneumovirus research. J Clin Virol 53:97–105. doi:10.1016/j.jcv.2011.10.002 22074934

[7] Sedeyn K, Schepens B, Saelens X. 2019. Respiratory syncytial virus nonstructural proteins 1 and 2: exceptional disrupters of innate immune responses. PLoS Pathog 15:e1007984. doi:10.1371/journal.ppat.1007984 31622448 PMC6797084

[8] Heim CJ, van den Hoogen BG, Dutch RE. 2025. Human metapneumovirus: understanding the molecular mechanisms and pathology of infection. J Virol 99:e0028425. doi:10.1128/jvi.00284-25 40981434 PMC12548460

[9] Soto JA, Gálvez NMS, Benavente FM, Pizarro-Ortega MS, Lay MK, Riedel C, Bueno SM, Gonzalez PA, Kalergis AM. 2018. Human metapneumovirus: mechanisms and molecular targets used by the virus to avoid the immune system. Front Immunol 9:2466. doi:10.3389/fimmu.2018.02466 30405642 PMC6207598

[10] Bergeron HC, Tripp RA. 2021. Immunopathology of RSV: an updated review. Viruses 13:2478. doi:10.3390/v13122478 34960746 PMC8703574

[11] Dong Y, Xie Z, Xu L. 2025. Receptors and host factors: key players in human metapneumovirus infection. Front Cell Infect Microbiol 15:1557880. doi:10.3389/fcimb.2025.1557880 40235933 PMC11996802

[12] Li A, Gong C, Wang L, Han Y, Kang L, Hu G, Cao J, Li M, Guan X, Luo M, Yu L, Li Y, Huang F, Gao GF, Wang Q. 2025. Epidemiological and phylogenetic characteristics of human metapneumovirus in Beijing, China, 2014–2024. Sig Transduct Target Ther 10. doi:10.1038/s41392-025-02377-7 PMC12417542 40921736

[13] Boivin G, Mackay I, Sloots TP, Madhi S, Freymuth F, Wolf D, Shemer-Avni Y, Ludewick H, Gray GC, LeBlanc E. 2004. Global genetic diversity of human metapneumovirus fusion gene. Emerg Infect Dis 10:1154–1157. doi:10.3201/eid1006.031097 15207075 PMC3323143

[14] Huck B, Scharf G, Neumann-Haefelin D, Puppe W, Weigl J, Falcone V. 2006. Novel human metapneumovirus sublineage. Emerg Infect Dis 12:147–150. doi:10.3201/eid1201.050772 16494734 PMC3291390

[15] Groen K, van Nieuwkoop S, Meijer A, van der Veer B, van Kampen JJA, Fraaij PL, Fouchier RAM, van den Hoogen BG. 2023. Emergence and potential extinction of genetic lineages of human metapneumovirus between 2005 and 2021. mBio 14:e0228022. doi:10.1128/mbio.02280-22 36507832 PMC9973309

[16] Elias-Warren A, Bennett JC, Iwu CD, Starita LM, Stone J, Capodanno B, Prentice R, Han PD, Acker Z, Grindstaff SB, Reinhart D, Logue JK, Wolf CR, Boeckh M, Kong K, Xie H, Kim G, Greninger AL, Perofsky AC, Viboud C, Uyeki TM, Englund JA, Roychoudhury P, Chu HY. 2025. Epidemiology of human metapneumovirus infection in a community setting, Seattle, Washington, USA. J Infect Dis 232:S78–S92. doi:10.1093/infdis/jiaf142 40668104 PMC12265066

[17] Skiadopoulos MH, Biacchesi S, Buchholz UJ, Riggs JM, Surman SR, Amaro-Carambot E, McAuliffe JM, Elkins WR, St. Claire M, Collins PL, Murphy BR. 2004. The two major human metapneumovirus genetic lineages are highly related antigenically, and the fusion (F) protein is a major contributor to this antigenic relatedness. J Virol 78:6927–6937. doi:10.1128/JVI.78.13.6927-6937.2004 15194769 PMC421687

[18] Papenburg J, Hamelin M-È, Ouhoummane N, Carbonneau J, Ouakki M, Raymond F, Robitaille L, Corbeil J, Caouette G, Frenette L, De Serres G, Boivin G. 2012. Comparison of risk factors for human metapneumovirus and respiratory syncytial virus disease severity in young children. J Infect Dis 206:178–189. doi:10.1093/infdis/jis333 22551815 PMC7114627

[19] Vicente D, Montes M, Cilla G, Perez-Yarza EG, Perez-Trallero E. 2006. Differences in clinical severity between genotype A and genotype B human metapneumovirus infection in children. Clin Infect Dis 42:e111–e113. doi:10.1086/504378 16705567

[20] Bosis S, Esposito S, Osterhaus ADME, Tremolati E, Begliatti E, Tagliabue C, Corti F, Principi N, Niesters HGM. 2008. Association between high nasopharyngeal viral load and disease severity in children with human metapneumovirus infection. J Clin Virol 42:286–290. doi:10.1016/j.jcv.2008.03.029 18479963 PMC7173119

[21] Moe N, Krokstad S, Stenseng IH, Christensen A, Skanke LH, Risnes KR, Nordbø SA, Døllner H. 2017. Comparing human metapneumovirus and respiratory syncytial virus: viral co-detections, genotypes and risk factors for severe disease. PLoS One 12:e0170200. doi:10.1371/journal.pone.0170200 28095451 PMC5240941

[22] Williams JV, Wang CK, Yang C-F, Tollefson SJ, House FS, Heck JM, Chu M, Brown JB, Lintao LD, Quinto JD, Chu D, Spaete RR, Edwards KM, Wright PF, Crowe JE Jr. 2006. The role of human metapneumovirus in upper respiratory tract infections in children: a 20-year experience. J Infect Dis 193:387–395. doi:10.1086/499274 16388486 PMC1586246

[23] Williams JV, Harris PA, Tollefson SJ, Halburnt-Rush LL, Pingsterhaus JM, Edwards KM, Wright PF, Crowe JE Jr. 2004. Human metapneumovirus and lower respiratory tract disease in otherwise healthy infants and children. N Engl J Med 350:443–450. doi:10.1056/NEJMoa025472 14749452 PMC1831873

[24] van den Hoogen BG, van Doornum GJJ, Fockens JC, Cornelissen JJ, Beyer WEP, de Groot R, Osterhaus ADME, Fouchier RAM. 2003. Prevalence and clinical symptoms of human metapneumovirus infection in hospitalized patients. J Infect Dis 188:1571–1577. doi:10.1086/379200 14624384

[25] Papenburg J, Boivin G. 2010. The distinguishing features of human metapneumovirus and respiratory syncytial virus. Rev Med Virol 20:245–260. doi:10.1002/rmv.651 20586081

[26] Wang X, Li Y, Deloria-Knoll M, Madhi SA, Cohen C, Ali A, Basnet S, Bassat Q, Brooks WA, Chittaganpitch M, et al.. 2021. Global burden of acute lower respiratory infection associated with human metapneumovirus in children under 5 years in 2018: a systematic review and modelling study. Lancet Glob Health 9:e33–e43. doi:10.1016/S2214-109X(20)30393-4 33248481 PMC7783516

[27] Kulkarni D, Cong B, Ranjini MJK, Balchandani G, Chen S, Liang J, González Gordon L, Sobanjo-Ter Meulen A, Wang X, Li Y, Osei-Yeboah R, Templeton K, Nair H. 2025. The global burden of human metapneumovirus-associated acute respiratory infections in older adults: a systematic review and meta-analysis. Lancet Healthy Longev 6:100679. doi:10.1016/j.lanhl.2024.100679 39954700

[28] Branche AR, Edwards KM. 2025. Review: knowledge gained and gaps in understanding in the 25 years since human metapneumovirus was first identified as a cause of human disease. J Infect Dis 232:S1–S9. doi:10.1093/infdis/jiaf187 40668102 PMC12265062

[29] Jobe NB, Rose E, Winn AK, Goldstein L, Schneider ZD, Silk BJ. 2025. Human metapneumovirus seasonality and co-circulation with respiratory syncytial virus — United States, 2014–2024. MMWR Morb Mortal Wkly Rep 74:182–187. doi:10.15585/mmwr.mm7411a1 40179043 PMC11970723

[30] Billard M-N, Wildenbeest JG, Kole R, Rodgers-Gray B, Fullarton J, Bont L. 2025. Post-pandemic dynamics of the global circulation of human metapneumovirus and respiratory syncytial virus. J Infect Dis 232:S10–S18. doi:10.1093/infdis/jiaf086 40668101 PMC12265059

[31] Piret J, Boivin G. 2022. Viral interference between respiratory viruses. Emerg Infect Dis 28:273–281. doi:10.3201/eid2802.211727 35075991 PMC8798701

[32] Piret J, Boivin G. 2024. The impact of trained immunity in respiratory viral infections. Rev Med Virol 34:e2510. doi:10.1002/rmv.2510 38282407

[33] Geiser J, Boivin G, Huang S, Constant S, Kaiser L, Tapparel C, Essaidi-Laziosi M. 2021. RSV and HMPV infections in 3D tissue cultures: mechanisms involved in virus-host and virus-virus interactions. Viruses 13:139. doi:10.3390/v13010139 33478119 PMC7835908

[34] García Acevedo M, Sánchez Códez MI, Peromingo Matute E, Galán Sánchez F, Delgado Martín B, Quiroga de Castro A, Fernández Puentes V, Lubián López S, Alonso Ojembarrena A. 2025. Impact of universal nirsevimab prophylaxis on clinical and ultrasound presentations of lower respiratory tract infections in hospitalized pediatric patients. Eur J Pediatr 184:621. doi:10.1007/s00431-025-06448-3 40960546

[35] Fourati S, Reslan A, Bourret J, Casalegno J-S, Rahou Y, Chollet L, Pillet S, Tremeaux P, Dossou NC, Gault E, et al.. 2025. Genotypic and phenotypic characterisation of respiratory syncytial virus after nirsevimab breakthrough infections: a large, multicentre, observational, real-world study. Lancet Infect Dis 25:301–311. doi:10.1016/S1473-3099(24)00570-X 39419046

[36] Mengual-Chuliá B, Launes C, Cazorla E, López-Lacort M, Muñoz-Quiles C, Mira-Iglesias A, Agüera M, Penela-Sánchez D, Garcés-Sánchez M, Fernández-García C, Cervera H, Carballido-Fernández M, Pineda-Caplliure A, Mollar-Maseres J, Shalabi-Benavent M, Sanz-Herrero F, Díez-Domingo J, Muñoz-Almagro C, Orrico-Sánchez A, López-Labrador FX, MEDIPRIM and VAHNSI Networks. 2026. Genomic surveillance of respiratory syncytial virus during 2023/2024 nirsevimab prophylaxis, Eastern Spain, early results. Clin Microbiol Infect:S1198-743X(26)00186-2. doi:10.1016/j.cmi.2026.04.003 41974354

[37] Widmer K, Griffin MR, Zhu Y, Williams JV, Talbot HK. 2014. Respiratory syncytial virus‐ and human metapneumovirus‐associated emergency department and hospital burden in adults. Influenza Resp Viruses 8:347–352. doi:10.1111/irv.12234 PMC3984605 24512531

[38] Akhras N, Weinberg JB, Newton D. 2010. Human metapneumovirus and respiratory syncytial virus: subtle differences but comparable severity. Infect Dis Rep 2:e12. doi:10.4081/idr.2010.e12 24470892 PMC3892583

[39] van der Bie S, Fluit RC, Neijzen T, Euser SM, van Gorp ECM, Kalpoe J, Souverein D, Snijders D, Du Toit J, Osterhaus A, Gommers D, van Lelyveld SFL, Goeijenbier M. 2026. Is clinical outcome pathogen related? Characteristics and outcomes of ICU patients with severe acute respiratory infections: focusing on respiratory syncytial virus, human metapneumovirus, influenza virus, and parainfluenza virus. J Intensive Care Med 41:512–522. doi:10.1177/08850666251375938 41026954 PMC13157522

[40] Kim CK, Choi J, Callaway Z, Kim HB, Chung JY, Koh Y-Y, Shin BM. 2010. Clinical and epidemiological comparison of human metapneumovirus and respiratory syncytial virus in seoul, Korea, 2003-2008. J Korean Med Sci 25:342–347. doi:10.3346/jkms.2010.25.3.342 20191030 PMC2826723

[41] Althouse BM, Flasche S, Toizumi M, Nguyen H-AT, Vo HM, Le MN, Hashizume M, Ariyoshi K, Anh DD, Rodgers GL, Klugman KP, Hu H, Yoshida L-M. 2021. Differences in clinical severity of respiratory viral infections in hospitalized children. Sci Rep 11:5163. doi:10.1038/s41598-021-84423-2 33664311 PMC7933285

[42] Ng DC-E, Liew C-H, Tan KK, Awang EHB, Nazri FNBA, Maran AKT, Mohan VAAC, Ramachandran D, Chok M, Teh CH, Mohamad Nor A, Baharuddin SB, Khoo EJ. 2024. Clinical comparison of HMPV and RSV infections in hospitalised Malaysian children: a propensity score matched study. Clin Respir J 18:e13747. doi:10.1111/crj.13747 38529669 PMC10964171

[43] Goldstein LA, Michaels MG, Salthouse A, Toepfer AP, Musa S, Hickey RW, Johnson M, Wang-Erickson AF, Weinberg GA, Szilagyi PG, Schlaudecker EP, Staat MA, Sahni LC, Boom JA, Klein EJ, Englund JA, Schuster JE, Selvarangan R, Harrison CJ, Halasa NB, Stewart LS, Dawood FS, Moline HL, Williams JV. 2025. Human metapneumovirus and respiratory syncytial virus in children: a comparative analysis. Pediatrics 156:e2024070218. doi:10.1542/peds.2024-070218 40763933 PMC12824835

[44] Azar B, Hashavya S, Ohana Sarna Cahan L, Reif S, Gross I. 2023. Bronchiolitis due to RSV and HMPV-epidemiology, clinical course, and prognosis: experience of a single tertiary center. Clin Pediatr (Phila) 62:1032–1039. doi:10.1177/00099228231151401 36744682

[45] Aminisani N, Wood T, Waite B, Seeds R, Jelley L, Wong C, Sue Huang Q. 2025. The impact of chronic medical conditions on the risk of human metapneumovirus hospitalizations in New Zealand adults, 2012–2015. J Infect Dis 232:S59–S68. doi:10.1093/infdis/jiaf226 40668093 PMC12265055

[46] Loubet P, Mathieu P, Lenzi N, Galtier F, Lainé F, Lesieur Z, Vanhems P, Duval X, Postil D, Amour S, Rogez S, Lagathu G, L’Honneur A-S, Foulongne V, Houhou N, Lina B, Carrat F, Launay O, Fluvac study group. 2021. Characteristics of human metapneumovirus infection in adults hospitalized for community-acquired influenza-like illness in France, 2012–2018: a retrospective observational study. Clin Microbiol Infect 27:127. doi:10.1016/j.cmi.2020.04.005 PMC7195031 32283266

[47] Sobanjo-ter Meulen A, Gutierrez AV, Ruiz O, Eeuwijk J, Vroling H, Kanesa-thasan N. 2025. The burden of human metapneumovirus (hMPV) disease in older and high-risk adults in developed countries: a systematic literature review. Infect Dis Ther 14:1917–1933. doi:10.1007/s40121-025-01187-2 40742420 PMC12339782

[48] Chan M, Koo SH, Jiang B, Lim PQ, Tan TY. 2018. Comparison of the biofire FilmArray respiratory panel, seegene AnyplexII RV16, and argene for the detection of respiratory viruses. J Clin Virol 106:13–17. doi:10.1016/j.jcv.2018.07.002 30007137 PMC7185839

[49] Chan W-S, Ho CW-Y, Chan T-C, Hung J, To M-Y, Leung S-M, Lai K-C, Wong C-Y, Leung C-P, Au C-H, Wan TS-K, Zee JS-T, Ma ES-K, Tang BS-F. 2024. Clinical evaluation of the biofire spotfire respiratory panel. Viruses 16:600. doi:10.3390/v16040600 38675941 PMC11055108

[50] Huang H-S, Tsai C-L, Chang J, Hsu T-C, Lin S, Lee C-C. 2018. Multiplex PCR system for the rapid diagnosis of respiratory virus infection: systematic review and meta-analysis. Clin Microbiol Infect 24:1055–1063. doi:10.1016/j.cmi.2017.11.018 29208560 PMC7128951

[51] Guo L, Li L, Liu L, Zhang T, Sun M. 2023. Neutralising antibodies against human metapneumovirus. Lancet Microbe 4:e732–e744. doi:10.1016/S2666-5247(23)00134-9 37499668

[52] Bhattacharyya S, Gesteland PH, Korgenski K, Bjørnstad ON, Adler FR. 2015. Cross-immunity between strains explains the dynamical pattern of paramyxoviruses. Proc Natl Acad Sci USA 112:13396–13400. doi:10.1073/pnas.1516698112 26460003 PMC4629340

[53] Rappazzo CG, Hsieh C-L, Rush SA, Esterman ES, Delgado T, Geoghegan JC, Wec AZ, Sakharkar M, Más V, McLellan JS, Walker LM. 2022. Potently neutralizing and protective anti-human metapneumovirus antibodies target diverse sites on the fusion glycoprotein. Immunity 55:1710–1724. doi:10.1016/j.immuni.2022.07.003 35944529

[54] Huang J, Diaz D, Mousa JJ. 2019. Antibody epitopes of pneumovirus fusion proteins. Front Immunol 10:2778. doi:10.3389/fimmu.2019.02778 31849961 PMC6895023

[55] Xiao X, Tang A, Cox KS, Wen Z, Callahan C, Sullivan NL, Nahas DD, Cosmi S, Galli JD, Minnier M, Verma D, Babaoglu K, Su H, Bett AJ, Vora KA, Chen Z, Zhang L. 2019. Characterization of potent RSV neutralizing antibodies isolated from human memory B cells and identification of diverse RSV/hMPV cross-neutralizing epitopes. MAbs 11:1415–1427. doi:10.1080/19420862.2019.1654304 31402751 PMC6816417

[56] Corti D, Bianchi S, Vanzetta F, Minola A, Perez L, Agatic G, Guarino B, Silacci C, Marcandalli J, Marsland BJ, Piralla A, Percivalle E, Sallusto F, Baldanti F, Lanzavecchia A. 2013. Cross-neutralization of four paramyxoviruses by a human monoclonal antibody. Nature 501:439–443. doi:10.1038/nature12442 23955151

[57] Schuster JE, Cox RG, Hastings AK, Boyd KL, Wadia J, Chen Z, Burton DR, Williamson RA, Williams JV. 2015. A broadly neutralizing human monoclonal antibody exhibits *in vivo* efficacy against both human metapneumovirus and respiratory syncytial virus. J Infect Dis 211:216–225. doi:10.1093/infdis/jiu307 24864121 PMC4342691

[58] Wen X, Suryadevara N, Kose N, Liu J, Zhan X, Handal LS, Williamson LE, Trivette A, Carnahan RH, Jardetzky TS, Crowe JE Jr. 2023. Potent cross-neutralization of respiratory syncytial virus and human metapneumovirus through a structurally conserved antibody recognition mode. Cell Host Microbe 31:1288–1300. doi:10.1016/j.chom.2023.07.002 37516111 PMC10527986

[59] Cabán M, Rodarte JV, Bibby M, Gray MD, Taylor JJ, Pancera M, Boonyaratanakornkit J. 2023. Cross-protective antibodies against common endemic respiratory viruses. Nat Commun 14:798. doi:10.1038/s41467-023-36459-3 36781872 PMC9923667

[60] Fausther-Bovendo H, Hamelin M-E, Carbonneau J, Venable M-C, Checkmahomed L, Lavoie P-O, Ouellet M-È, Boivin G, D’Aoust M-A, Kobinger GP. 2022. A candidate therapeutic monoclonal antibody inhibits both HRSV and HMPV replication in mice. Biomedicines 10:2516. doi:10.3390/biomedicines10102516 36289776 PMC9599547

[61] Zhai H, Yu W, Wang J, Deng J, Lei S, Zhou T, Li Y, Xu K, Ma M, Feng R, Hu Y, Ren L, Cao Y, Liu E, Wang X. 2026. Antibody cocktails based on the occupationally acquired immunity of pediatricians neutralize and confer protection against RSV and hMPV. Sci Transl Med 18:eadz4170. doi:10.1126/scitranslmed.adz4170 41706868

[62] Williams JV, Chen Z, Cseke G, Wright DW, Keefer CJ, Tollefson SJ, Hessell A, Podsiad A, Shepherd BE, Sanna PP, Burton DR, Crowe JE Jr, Williamson RA. 2007. A recombinant human monoclonal antibody to human metapneumovirus fusion protein that neutralizes virus *in vitro* and is effective therapeutically *in vivo*. J Virol 81:8315–8324. doi:10.1128/JVI.00106-07 17522220 PMC1951312

[63] Wen X, Krause JC, Leser GP, Cox RG, Lamb RA, Williams JV, Crowe JE Jr, Jardetzky TS. 2012. Structure of the human metapneumovirus fusion protein with neutralizing antibody identifies a pneumovirus antigenic site. Nat Struct Mol Biol 19:461–463. doi:10.1038/nsmb.2250 22388735 PMC3546531

[64] Ulbrandt ND, Ji H, Patel NK, Riggs JM, Brewah YA, Ready S, Donacki NE, Folliot K, Barnes AS, Senthil K, Wilson S, Chen M, Clarke L, MacPhail M, Li J, Woods RM, Coelingh K, Reed JL, McCarthy MP, Pfarr DS, Osterhaus ADME, Fouchier RAM, Kiener PA, Suzich JA. 2006. Isolation and characterization of monoclonal antibodies which neutralize human metapneumovirus *in vitro* and *in vivo*. J Virol 80:7799–7806. doi:10.1128/JVI.00318-06 16873237 PMC1563801

[65] Hamelin M-E, Gagnon C, Prince GA, Kiener P, Suzich J, Ulbrandt N, Boivin G. 2010. Prophylactic and therapeutic benefits of a monoclonal antibody against the fusion protein of human metapneumovirus in a mouse model. Antiviral Res 88:31–37. doi:10.1016/j.antiviral.2010.07.001 20619294

[66] Wu S-J, Schmidt A, Beil EJ, Day ND, Branigan PJ, Liu C, Gutshall LL, Palomo C, Furze J, Taylor G, Melero JA, Tsui P, Del Vecchio AM, Kruszynski M. 2007. Characterization of the epitope for anti-human respiratory syncytial virus F protein monoclonal antibody 101F using synthetic peptides and genetic approaches. J Gen Virol 88:2719–2723. doi:10.1099/vir.0.82753-0 17872524

[67] McLellan JS, Chen M, Chang J-S, Yang Y, Kim A, Graham BS, Kwong PD. 2010. Structure of a major antigenic site on the respiratory syncytial virus fusion glycoprotein in complex with neutralizing antibody 101F. J Virol 84:12236–12244. doi:10.1128/JVI.01579-10 20881049 PMC2976384

[68] Más V, Rodriguez L, Olmedillas E, Cano O, Palomo C, Terrón MC, Luque D, Melero JA, McLellan JS. 2016. Engineering, structure and immunogenicity of the human metapneumovirus F protein in the postfusion conformation. PLoS Pathog 12:e1005859. doi:10.1371/journal.ppat.1005859 27611367 PMC5017722

[69] González AE, Lay MK, Jara EL, Espinoza JA, Gómez RS, Soto J, Rivera CA, Abarca K, Bueno SM, Riedel CA, Kalergis AM. 2017. Aberrant T cell immunity triggered by human Respiratory Syncytial Virus and human Metapneumovirus infection. Virulence 8:685–704. doi:10.1080/21505594.2016.1265725 27911218 PMC5626346

[70] Schmidt ME, Varga SM. 2018. The CD8 T cell response to respiratory virus infections. Front Immunol 9:678. doi:10.3389/fimmu.2018.00678 29686673 PMC5900024

[71] Harris ED, McGovern M, Pernikoff S, Ikeda R, Kipnis L, Hannon W, Sobolik EB, Gray M, Greninger AL, He S, Chin C-N, Fu T-M, Pancera M, Boonyaratanakornkit J. 2026. Development of a potent monoclonal antibody for treatment of human metapneumovirus infections. Nat Commun 17:2714. doi:10.1038/s41467-026-69328-w 41680141 PMC13013820

[72] Castro KM, Watson JL, Wang J, Southern J, Ayardulabi R, Georgeon S, Rosset S, Baker D, Correia BE. 2026. Accurate single-domain scaffolding of three nonoverlapping protein epitopes using deep learning. Nat Chem Biol 22:604–611. doi:10.1038/s41589-025-02083-z 41350440 PMC13038408

[73] Gnanasekaran S, Bashar MA, Rajan AK, Prabhat P. 2025. Emerging threat of human metapneumovirus (HMPV) and strategies for its containment and control. Infect Genet Evol 131:105758. doi:10.1016/j.meegid.2025.105758 40345565

[74] Nikiforow S, Horowitz M, McCarty JM, Abbas A, Coghill J, Stanton J, Ma J, Vasileiou S, Gilmore S, Dholaria DrB, Hanna R, Taplitz RA, Bansal R. 2024. ALVR106, an off-the-shelf, multivirus-specific T-cell therapy, for the treatment of respiratory viral infections: results from a phase 1, first-in-human, dose-ranging trial. Transplant Cell Ther 30:S83–S84. doi:10.1016/j.jtct.2023.12.135

[75] Krüger N, Laufer SA, Pillaiyar T. 2025. An overview of progress in human metapneumovirus (hMPV) research: Structure, function, and therapeutic opportunities. Drug Discov Today 30:104364. doi:10.1016/j.drudis.2025.104364 40286981

[76] Principi N, Fainardi V, Esposito S. 2025. Human metapneumovirus: a narrative review on emerging strategies for prevention and treatment. Viruses 17:1140. doi:10.3390/v17081140 40872853 PMC12390667

[77] Safdar A. 2008. Immune modulatory activity of ribavirin for serious human metapneumovirus disease: early i.v. therapy may improve outcomes in immunosuppressed SCT recipients. Bone Marrow Transplant 41:707–708. doi:10.1038/bmt.2008.80 18347567

[78] Shachor-Meyouhas Y, Ben-Barak A, Kassis I. 2011. Treatment with oral ribavirin and IVIG of severe human metapneumovirus pneumonia (HMPV) in immune compromised child. Pediatr Blood Cancer 57:350–351. doi:10.1002/pbc.23019 21671370

[79] Beaird OE, Freifeld A, Ison MG, Lawrence SJ, Theodoropoulos N, Clark NM, Razonable RR, Alangaden G, Miller R, Smith J, Young JAH, Hawkinson D, Pursell K, Kaul DR. 2016. Current practices for treatment of respiratory syncytial virus and other non-influenza respiratory viruses in high-risk patient populations: a survey of institutions in the Midwestern Respiratory Virus Collaborative. Transpl Infect Dis 18:210–215. doi:10.1111/tid.12510 26923867 PMC7169710

[80] Shahda S, Carlos WG, Kiel PJ, Khan BA, Hage CA. 2011. The human metapneumovirus: a case series and review of the literature. Transplant Infectious Dis 13:324–328. doi:10.1111/j.1399-3062.2010.00575.x PMC3107511 21631655

[81] Knowles SR, Phillips EJ, Dresser L, Matukas L. 2003. Common adverse events associated with the use of ribavirin for severe acute respiratory syndrome in Canada. Clin Infect Dis 37:1139–1142. doi:10.1086/378304 14523782 PMC7204073

[82] Kim HW, Canchola JG, Brandt CD, Pyles G, Chanock RM, Jensen K, Parrott RH. 1969. Respiratory syncytial virus disease in infants despite prior administration of antigenic inactivated vaccine. Am J Epidemiol 89:422–434. doi:10.1093/oxfordjournals.aje.a120955 4305198

[83] Ison MG, Papi A, Athan E, Feldman RG, Langley JM, Lee D-G, Leroux-Roels I, Martinon-Torres F, Schwarz TF, van Zyl-Smit RN, et al.. 2024. Efficacy and safety of respiratory syncytial virus (RSV) prefusion F protein vaccine (RSVPreF3 OA) in older adults over 2 RSV seasons. Clin Infect Dis 78:1732–1744. doi:10.1093/cid/ciae010 38253338 PMC11175669

[84] Wilson E, Goswami J, Baqui AH, Doreski PA, Perez-Marc G, Zaman K, Monroy J, Duncan CJA, Ujiie M, Rämet M, et al.. 2023. Efficacy and safety of an mRNA-based RSV PreF vaccine in older adults. N Engl J Med 389:2233–2244. doi:10.1056/NEJMoa2307079 38091530

[85] Walsh EE, Pérez Marc G, Zareba AM, Falsey AR, Jiang Q, Patton M, Polack FP, Llapur C, Doreski PA, Ilangovan K, et al.. 2023. Efficacy and safety of a bivalent RSV prefusion F vaccine in older adults. N Engl J Med 388:1465–1477. doi:10.1056/NEJMoa2213836 37018468

[86] Hsieh C-L, Rush SA, Palomo C, Chou C-W, Pickens W, Más V, McLellan JS. 2022. Structure-based design of prefusion-stabilized human metapneumovirus fusion proteins. Nat Commun 13:1299. doi:10.1038/s41467-022-28931-3 35288548 PMC8921277

[87] Pilaev M, Shen Y, Carbonneau J, Venable M-C, Rhéaume C, Lavigne S, Couture C, Guarné A, Hamelin M-È, Boivin G. 2020. Evaluation of pre- and post-fusion Human metapneumovirus F proteins as subunit vaccine candidates in mice. Vaccine 38:2122–2127. doi:10.1016/j.vaccine.2020.01.047 32007293

[88] Saul S, Dahal B, Luongo C, Liu X, Yang L, Santos C, Dai J, Zhang L, Buchholz UJ, Munir S. 2025. Intranasal bovine/human parainfluenza virus 3 vaccine candidates expressing human metapneumovirus wild-type or pre-fusion F protein elicit protective immunity against human metapneumovirus in hamsters. J Virol 99:e0114525. doi:10.1128/jvi.01145-25 41288380 PMC12724368

[89] Yim KC, Cragin RP, Boukhvalova MS, Blanco JCG, Hamlin M-E, Boivin G, Porter DD, Prince GA. 2007. Human metapneumovirus: enhanced pulmonary disease in cotton rats immunized with formalin-inactivated virus vaccine and challenged. Vaccine 25:5034–5040. doi:10.1016/j.vaccine.2007.04.075 17543425 PMC1937335

[90] Hamelin M-È, Couture C, Sackett MK, Boivin G. 2007. Enhanced lung disease and Th2 response following human metapneumovirus infection in mice immunized with the inactivated virus. J Gen Virol 88:3391–3400. doi:10.1099/vir.0.83250-0 18024909

[91] Karron RA, San Mateo J, Wanionek K, Collins PL, Buchholz UJ. 2018. Evaluation of a live attenuated human metapneumovirus vaccine in adults and children. J Pediatric Infect Dis Soc 7:86–89. doi:10.1093/jpids/pix006 28444226 PMC6075531

[92] August A, Shaw CA, Lee H, Knightly C, Kalidindia S, Chu L, Essink BJ, Seger W, Zaks T, Smolenov I, Panther L. 2022. Safety and immunogenicity of an mRNA-based human metapneumovirus and parainfluenza virus type 3 combined vaccine in healthy adults. Open Forum Infect Dis 9:ofac206. doi:10.1093/ofid/ofac206 35794943 PMC9251669

[93] Schnyder Ghamloush S, Essink B, Hu B, Kalidindi S, Morsy L, Egwuenu-Dumbuya C, Kapoor A, Girard B, Dhar R, Lackey R, Snape MD, Shaw CA. 2024. Safety and immunogenicity of an mRNA-Based hMPV/PIV3 combination vaccine in seropositive children. Pediatrics 153:e2023064748. doi:10.1542/peds.2023-064748 38738290

[94] FDA/VRBPAC. 2024. Considerations for respiratory syncytial virus (RSV) vaccine safety in pediatric populations. Available from: https://www.fda.gov/media/184301/download. Retrieved 27 Mar 2026.

[95] Vicebio. 2025. Pipeline targeting respiratory viruses. Available from: https://vicebio.com/pipeline/

[96] Clover. 2026. Clover initiates phase 2 clinical trial for RSV + hMPV ± PIV3 respiratory combination vaccine candidates. Available from: https://www.cloverbiopharma.com/media/189.html. Retrieved 27 Mar 2026.

[97] Clover. 2025. Clover initiates phase I clinical trial for RSV + hMPV ± PIV3 respiratory combination vaccine candidates. Available from: https://www.cloverbiopharma.com/media/183.html. Retrieved 27 Mar 2026.

[98] Shapiro C, Sánchez-Crespo N, Ciarlet M, Hourguettes N, Wen J, Rida W, Price J, Engram AE, Adams EM, Kanesa-Thasan N. 2025. A randomized phase 1 clinical trial of a respiratory syncytial virus and human metapneumovirus combination protein-based virus-like particle vaccine in adults 60-75 years of age. Open Forum Infect Dis 12:ofaf160. doi:10.1093/ofid/ofaf160 40201719 PMC11977332

[99] Ruckel CE, Wolf JD, Plemper RK. 2025. Status of advanced respiratory syncytial virus antiviral therapeutics 2025. Curr Opin Virol 73:101477. doi:10.1016/j.coviro.2025.101477 40848680 PMC13526433

[100] Zhang H, Zhao W, Zhang X, Zhang L, Guo R, Huang H, Lin L, Liu F, Chen H, Shen F, et al.. 2025. Efficacy and safety of ziresovir in hospitalised infants aged 6 months or younger with respiratory syncytial virus infection in China: findings from a phase 3 randomised trial with 24-month follow-up. Lancet Child Adolescent Health 9:325–336. doi:10.1016/S2352-4642(25)00067-7 40246359

[101] hVIVO. 2026. HMPV human challenge model. Available from: https://hvivo.com/human-challenge-models/hmpv/. Retrieved 27 Mar 2026.

